# A systematic review exploring the link between chronic venous disease and neuropathy

**DOI:** 10.1016/j.jvsv.2026.102602

**Published:** 2026-08-19

**Authors:** Vritika Ravisangar, Jessica Bowie, Marwah Salih, Matthew Tan, Rachel Low, Sarah Onida, Alun H. Davies

**Affiliations:** Section of Vascular Surgery, Department of Surgery and Cancer, Imperial College London, London, UK

**Keywords:** Chronic venous disease, Peripheral neuropathy, Sensory neuropathy, Venous leg ulcer

## Abstract

**Objective:**

Patients with chronic venous disease (CVD) often experience pain and heaviness; however, many report additional neuropathic symptoms, such as burning, tingling, and decreased sensations. Despite these occurrences, the nature and pathophysiology of neuropathy in patients with CVD are unclear. The objective of this systematic review was to estimate the prevalence and features of neuropathy in patients with CVD, identify methods for assessing neuropathy in this population, and explore potential pathophysiological mechanisms underlying its development.

**Methods:**

This systematic review was performed according to the Preferred Reporting Items for Systematic Reviews and Meta-Analyses guidelines, with the protocol registered on International Prospective Register of Systematic Reviews (PROSPERO; CRD420251124308). The MEDLINE, EMBASE, and Cochrane CENTRAL databases were searched from 1947 to August 2025. The eligibility criteria included studies examining patients with CVD who underwent objective neuropathy assessments. Studies involving patients with comorbidities predisposing them to peripheral neuropathy were excluded. Studies without objective neuropathy assessments were also excluded.

**Results:**

Of 2313 articles retrieved from the search, eight articles were included in this review. The majority of studies investigated sensory neuropathy. The most common objective neuropathy assessment methods were testing vibration detection thresholds, which was performed in five studies, and temperature discrimination, which was performed in four studies. Other assessment methods included the Neuropathy Disability Score, nerve conduction studies, monofilament testing, tendon reflex testing, and skin biopsies. Various nerve fibers responsible for different functions were affected, including A-alpha, A-beta, A-delta, and C-fibers. Furthermore, patients with more severe CVD (C5-C6) had significantly worse neuropathy.

**Conclusions:**

The included studies reported neuropathy prevalence ranging from 33% to 100%; however, direct comparability was greatly limited by heterogeneous assessment methods and the lack of pooled confidence intervals. There was evidence of both sensory and motor neuropathy in patients with CVD. Potential pathophysiological mechanisms underlying neuropathy in patients with CVD include venous hypertension translating to increased endoneurial pressure and ischemia, venous microangiopathy, and axonal hypoxia. Sensory impairment due to nerve injury associated with CVD may increase the risk of skin trauma potentially leading to venous leg ulceration. This systematic review underscores the potential association between neuropathy and patients with CVD and suggest that neuropathy may be associated with more severe CVD.

**Article Highlights**

• **Type of Research:** Systematic review.
• **Key Findings:** Summary of the available literature identified a potential association between peripheral neuropathy and chronic venous disease, with neuropathy appearing to be more severe in patients with advanced chronic venous disease. However, substantial heterogeneity in neuropathy assessment methods and study populations limited direct comparison between studies.
• **Take Home message:** Peripheral neuropathy may be an under recognized occurrence in chronic venous disease, particularly in more advanced disease. Further research using standardized neuropathy assessment methods is needed to clarify this association and determine its clinical significance.

Chronic venous disease (CVD) is a prevalent condition, ranging from 45.6% to 83.6% of adults being affected by Clinical-Etiology-Anatomy-Pathophysiology (CEAP) classes C0 to C6 globally.^1^ It presents a large economic burden on the National Health Service; the cost of managing an unhealed venous leg ulcer (VLU) is estimated to be £13,500.^2^ In the United States, it is estimated to cost $34,000 or more to treat a chronic, nonhealing venous ulcer.^3^ VLU predominantly affects older people and can have debilitating repercussions, severely impacting their daily lives by causing inability to work and reducing psychosocial functioning due to pain and restricted mobility.^1^^,^^4^ Patients can often experience a range of symptoms, including cramping, heaviness, and swelling, as well as skin changes that can ultimately lead to the most severe manifestation of active venous ulceration. Risk factors for developing CVD include older age, genetic predisposition, pregnancy, obesity, physical inactivity, and prolonged sitting or standing.^5^

CVD develops from valvular failure leading to reflux or venous outflow obstruction, with both mechanisms causing venous hypertension.^6^ Many patients report additional symptoms such as burning, paresthesia, and decreased sensation that are suggestive of peripheral neuropathy, a condition where peripheral nerves are damaged.^7^^,^^8^ Unlike nociceptive pain, neuropathic pain can occur without an identifiable, obvious cause, which means that the nature and pathophysiology of neuropathy in CVD is still unclear.^9^ Venous hypertension may cause venous microangiopathy and impaired blood flow to nerves, leading to ischemia and hypoxia.^10^ In addition, this can cause edema in nerve fascicles, compressing their axons, which also leads to nerve dysfunction and neuropathy.^7^ Other hypotheses include inflammatory factors creating pericapillary fibrin cuffs that obstruct oxygenation of nerves, and depletion of neuropeptides from excessive sympathetic activation consequent vasoconstriction.^7^^,^^11^

Establishing the essence of this association is beneficial as patients presenting with venous-related neurological symptoms should not be dismissed. The more CVD deteriorates, the worse the patient’s quality of life becomes, as shown by the correlation with increasing CEAP class and Venous Disability Score.^12^, ^13^, ^14^ Improving awareness of neuropathy in CVD could aid in understanding the disease process, advising pain management regimens as conservative management and lifestyle modification can fail to achieve intended effects.^15^ Therefore, it is in the best interest of the patient to understand this association to help early prevention of the disease and reduce the risk of injury in the presence of neuropathy.^12^

This systematic review aims to estimate the prevalence of neuropathy in patients with CVD, identify the methods of assessing this, explore potential pathophysiological mechanisms of neuropathy development in venous disease, and to provide a qualitative summary of the included study findings.

## Methods

### Search strategy

The MEDLINE, EMBASE, and Cochrane CENTRAL databases were searched up to August 15, 2025 using keywords such as “venous disease,” “venous insufficiency,” “peripheral neuropathy,” and “nerve damage.” Full search strategies for each database, dates accessed, and number of results produced for each database are available in the Supplementary Material (online only).

### Inclusion and exclusion criteria

The inclusion criteria included: studies recruiting adults aged 18 years or older, studies of participants with a clinical diagnosis of CVD based on either clinical assessment, duplex ultrasound guidance, or CEAP classification, studies where the outcome reported was any form of peripheral neuropathy (such as sensory, motor, and autonomic), and studies where peripheral neuropathy was objectively assessed. The study designs included were case series, single-arm studies, cross-sectional, case-control, cohort studies, and randomized controlled trials. The exclusion criteria included reporting of neuropathy based solely on nonspecific, subjective lower limb symptoms such as “ache” or “pain”; editorials; case reports; systematic reviews; meta-analyses; conference abstracts; animal studies; and studies not reported in English. In addition, studies that included patients with other conditions associated with peripheral neuropathy other than CVD, such as diabetes mellitus, autoimmune disease, human immunodeficiency virus, shingles, syphilis, vitamin B12 deficiency or stroke, were excluded to minimize confounding.

### Data screening

Title, abstract, and full-text screening were carried out on Covidence by three independent reviewers (V.R., J.B., and M.S.). Where there were conflicts on study inclusion, the reviewers discussed to come to a consensus, and in cases where this was not possible, a fourth reviewer (M.T.) decided on the study's inclusion.

### Data extraction

Three reviewers carried out data extraction from the included studies using a dedicated Excel spreadsheet. Data collected included country, study design, participant characteristics, CVD inclusion criteria from study, CEAP classification of the participants, method of neuropathy assessment, affected nerve fibers, and overall findings from the study.

### Data synthesis

A qualitative synthesis was carried out as there was heterogeneity between the included studies with their study designs, neuropathy assessment methods, and the reported outcomes, which meant a meta-analysis was not feasible.

Clinical heterogeneity was assessed by looking at differences in the population, the exposure, and the outcomes, as shown in Table I. There were high levels of heterogeneity in patient demographic, method of CVD diagnosis, exclusion criteria, and outcome reporting as shown in Table I. Many studies had residual confounding and potential confounders were rarely adjusted for. For example, only two studies required their patients to wear compression stockings before neuropathy assessment. The anatomical site of neuropathy assessment in studies varied, but was overall limited to the foot, gaiter areas, and thigh. There was no healthy control group for comparison in all studies. Therefore, methodological heterogeneity was consistently proven across included studies. These disparate features between the studies suggest that making direct comparisons between them would be inappropriate and therefore led to the decision to not conduct a meta-analysis.

**Table I:** Summary of clinical and methodological heterogeneity across included studies

Studies	Countries	Study design	Population (CEAP class)	Sample size	Age	Sex (% male)	CVD confirmation method	Types of neuropathy	Assessment method	Anatomical site tested	Outcome metric reported	Overall risk of bias (tool)	Overall comparability with other studies
Arseculeratne et al^16^	England	Cross-Sectional	C5/C6 (n = 15)	15	77 (mean)	Not stated	Clinical diagnosis	Sensory	10-g Owen Mumford monofilament	Plantar and dorsal foot	% of patients with sensory loss	High (JBI)	Low
Gozke et al^8^	Turkey	Case Control	C1-C4 (n = 35)	35	54.5 (mean)	48.6	Venous Doppler ultrasound, physical examination, dermal manifestations	Motor and sensory	Electromyography (NCS): peroneal CMAP, tibial MCV, sural SNAP	Head of fibula (peroneal nerve), ankle and popliteal (peroneal), lateral malleoli and triceps surae (sural)	CMAP amplitude (mV), MCV (m/s), SNAP detectability (%)	Moderate (ROBINS-I)	Moderate
Guest et al^17^	England	Cross Sectional	C5 (n = 14) C6 (n = 15)	29	75 (mean)	28	Duplex scanning	Peripheral	Punch biopsy with immunostaining for PGP 9.5, CGRP, Substance P, and CD34. Fiber quantification	Skin adjacent to and proximal to venous ulcer	Fiber number, fiber length (μm), fiber density	High (JBI)	Low
Newland et al^18^	The United States	Case Control (pilot)	C5/C6 (n = 10)	10	Not stated	Not stated	Clinical diagnosis, CEAP classification	Sensory	NDS: 10-g monofilament, vibration perception (128 Hz tuning fork), temperature discrimination, Achilles tendon reflex	Apex of first toe, dorsum of foot, back of ankle	Composite NDS	Moderate (ROBINS-I)	Moderate
Padberg et al^19^	The United States	Cross-sectional	C2 (47.8%) C5 (52.2%)	14	Not stated	100	Duplex scanning, air plethysmography	Sensory	Deep tendon reflexes, vibratory sensation (biothesiometer), proprioception, light touch. Motor strength also tested	Five sites: proximal to medial malleolus, proximal to lateral malleolus, proximal medial calf, proximal lateral calf, thigh	Sensory threshold values at each site	Moderate (JBI)	Moderate
Reinhardt et al^7^	Germany	Case control	C4 (n = 6) C5/C6 (n = 24)	30	55.2 (mean)	47	Venous Doppler ultrasound, clinical examination, phlebography	Motor and Sensory	Motor and sensory NCS, vibration testing, thermotesting, Quantitative Sudomotor Axon Reflex Test (QSAT)	Peroneal nerve (distal motor latency), warm/cold detection thresholds	DML (ms), warm/cold detection thresholds (°C), vibration sense	Moderate (ROBINS-I)	Moderate
Shami et al^11^	England	Case control	Not stated	40	65 (median)	52.5	Duplex scanning	Sensory	Thresholds to warming, cooling, and vibration	Not stated	Threshold values to warming/cooling (°C), vibration	Moderate (ROBINS-I)	Moderate
Yim et al^10^	The United States	Cross-sectional	C1 (22%) C2 (34%) C5 (6%) C6 (38%)	42	64 (mean)	33	Clinical diagnosis, CEAP classification	Sensory	NDS: 10-g monofilament, vibration perception threshold, temperature discrimination, Achilles tendon reflex examination	Not specified beyond foot	Composite NDS	Moderate (JBI)	Moderate

*CEAP*, Clinical-Etiological-Anatomy-Pathophysiology; *CD34*, cluster determinant 34; *CGRP*, calcitonin gene-related peptide; *CMAP*, compound muscle action potential; *CVD*, chronic venous disease; *DML*, distal motor latency; *JBI*, Joanna Briggs Institute; *MCV*, motor conduction velocity; *NCS*, nerve conduction studies; *NDS*, Neuropathy Disability Score; *PGP*, protein gene product; *ROBINS-I*, Risk Of Bias In Non-randomized Studies—of Interventions; *SNAP*, sensory nerve action potential.

Owing to the dissimilar and noncomparable outcome measures, a formal statistical heterogeneity assessment could not be carried out using the I^2^ statistic.

For a meaningful random-effects model, we aimed to consider subgroups with at least three studies, therefore Neuropathy Disability Score (NDS), nerve conduction studies (NCS), monofilament testing, or immunohistochemistry subgroups could not be considered. There were three studies in the vibration testing subgroup. The instrument used to measure vibration perception in each study differed between using a biothesiometer and a 128-Hz tuning fork. The studies did not report the outcome in the same units, as one study reported the presence of vibration sense on a scale of 0 to 3, whereas the other two studies reported raw vibration threshold values. The CEAP classes that one of the studies investigated was not stated, the CEAP distributions across the remaining studies were notably varied. Furthermore, one of the studies only looked at male participants, so the populations of these studies are not comparable. Because of these factors, pooling was not feasible.

### Risk of bias assessment

The quality of the included studies was assessed using the Risk Of Bias In Non-randomized Studies—of Interventions (ROBINS-I) tool for nonrandomized case-control studies and cross-sectional studies, the Joanna Briggs Institute Critical Appraisal Checklist for Analytical Cross-Sectional Studies was used.^20^ ROBINS-I was selected for case-control studies as this is the standard tool recommended by Cochrane for nonrandomized studies comparing a health outcome between an exposed and unexposed group.^21^ The full details of all domain assessments are provided in the Supplementary Tables I and II (online only).

## Results

### Study screening and selection

**Fig 1:**
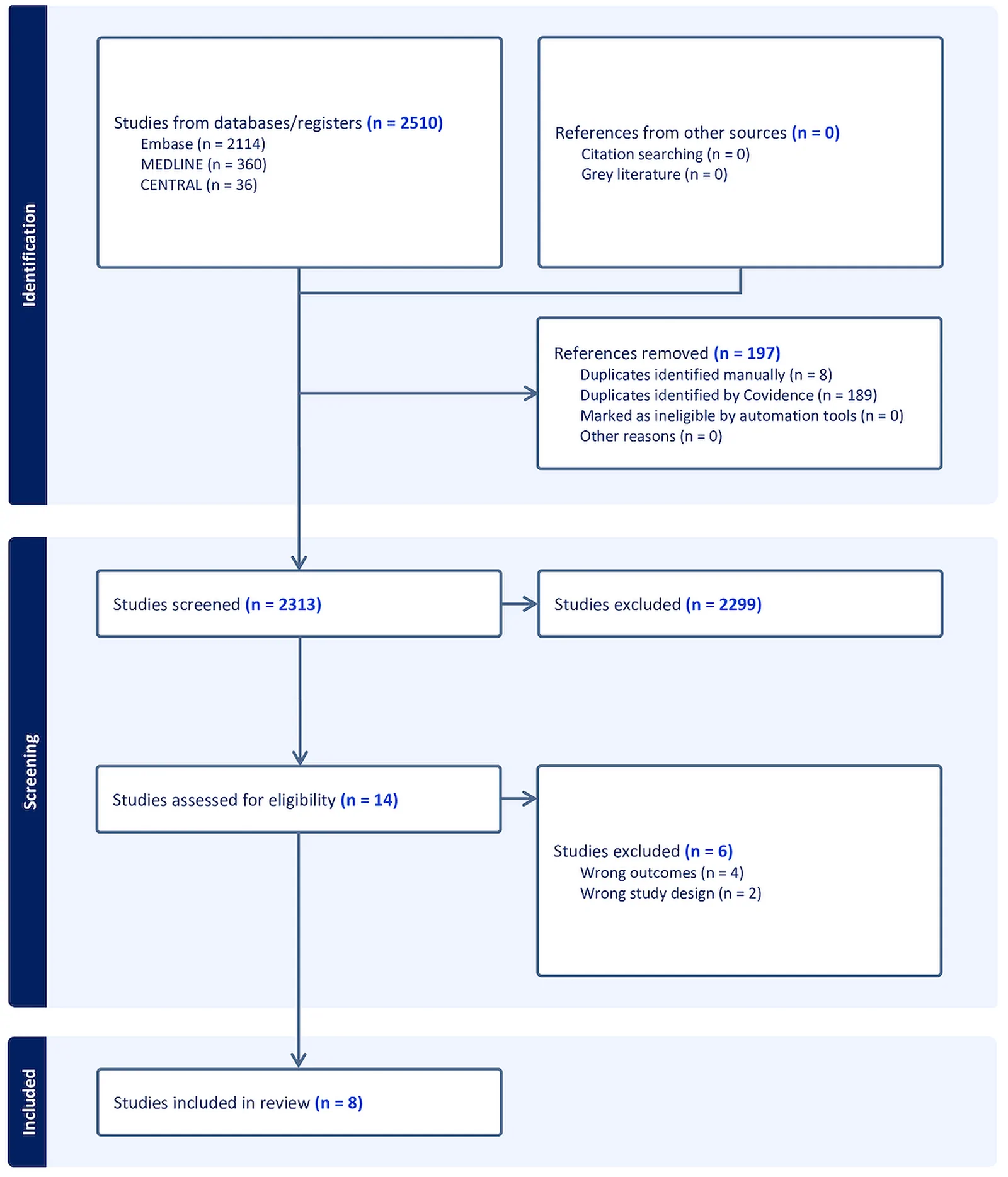
Preferred Reporting Items for Systematic Reviews and Meta-Analyses (PRISMA) flow diagram.

Of the 2313 articles after the removal of duplicates, 2299 articles were removed during title and abstract screening. Of the 14 articles that underwent full-text screening, six were excluded: four studies reported the wrong outcome, and two articles had ineligible study designs. Eight studies were therefore included in the narrative synthesis. Fig 1 summarizes the study selection process.

### Study characteristics

Study characteristics are outlined in Table II. There were four cross-sectional studies and four case-control studies; two of the papers were pilot studies (one cross-sectional and one case control study). The sample sizes of the included studies ranged from 10 to 42 patients, which were notably small and likely underpowered, therefore requiring a cautious interpretation of findings. The mean ages of the patients in the studies ranged from 54 to 77 years old. Seven studies investigated both male and female participants, the remaining study only included males. As part of their neuropathy assessment five studies included vibration testing, four studies looked at temperature perception, four studies performed deep tendon reflex examinations, three studies included a monofilament test, two papers performed NCS, one study a Quantitative Sudomotor Axon Reflex Test, and one study used punch biopsies and immunohistochemistry.

**Table II:** Characteristics of included studies

Studies	Countries	Study design	No. of patients	Age	Sex distribution (% male)	Chronic venous disease investigations	CEAP stage	Comorbidities excluded	Compression use	Interventions
Arseculeratne et al^16^	England	Cross-sectional	15	77 (mean)	Not stated	Clinical diagnosis	C5/C6: 15	Not stated	No	None
Gozke et al^8^	Turkey	Case control	35	54.5 (mean)	48.6	Venous Doppler ultrasound, physical examination documenting venous engorgement, edema, and dermal manifestations	C1-C4: 35	Etiologies other than CVD that might lead to neuropathy (exact diseases were not stated). Leg edema (compression stockings worn 6 hours before testing)	Yes–Worn before neurophysiological examinations	None
Guest et al^17^	England	Cross-sectional	29	75 (mean)	28	Duplex scanning	C5: 14 C6: 15	Patients with ulcers of etiologies other than venous	No	None
Newland et al^18^	The United States	Case control	10	Not stated	Not stated	Clinical diagnosis, CEAP classification	C5/C6: 10	Patients with a history of neuropathy or predisposing conditions for neuropathy	No	None
Padberg et al^19^	The United States	Cross-sectional	14	Not stated	100	Duplex scanning, air plethysmography	C2: 47.8% C5: 52.2%	Diabetes mellitus, ethanol abuse, vitamin deficiencies, limbs with previous saphenectomy or open fracture reductions.	No	None
Reinhardt et al^7^	Germany	Case control	30	55.2 (mean)	47	Venous Doppler ultrasound, clinical examination, in some cases, examination with conventional phlebography	C4: 6 C5/C6: 24	Diabetes mellitus, peripheral degenerative arteriopathy, coexistence of any conditions predisposing to peripheral neuropathy (exact diseases were not stated). Leg edema (compression stockings worn before testing).	Yes–Worn before neurophysiological examinations	None
Shami et al^11^	England	Case control	40	65 (median)	52.5	Duplex scanning	Not stated	Conditions associated with peripheral neuropathy	No	None
Yim et al^10^	The United States	Cross-sectional	42	64 (mean)	33	Clinical diagnosis, CEAP classification	C1: 22% C2: 34% C5: 6% C6: 38%	Patients at risk of peripheral neuropathy from other diseases such as: uncontrolled diabetes, HIV, vitamin B12 deficiency, shingles, autoimmune disease, Guillain-Barre syndrome, syphilis, family history of inherited neuropathy or myopathy, stroke, Hansen disease, cellulitis, BMI greater than 40 kg/m^2^, congestive heart failure, peripheral arterial disease, ankle brachial index less than 0.9, patients with restricted mobility secondary to pain, rheumatoid arthritis, osteoarthritis, patients who were immobile and restricted to a wheelchair	No	None

*BMI*, Body mass index; *CEAP*, Clinical-Etiological-Anatomy-Pathophysiology; *HIV*, human immunodeficiency virus.

### Quality assessment of studies

**Fig 2:**
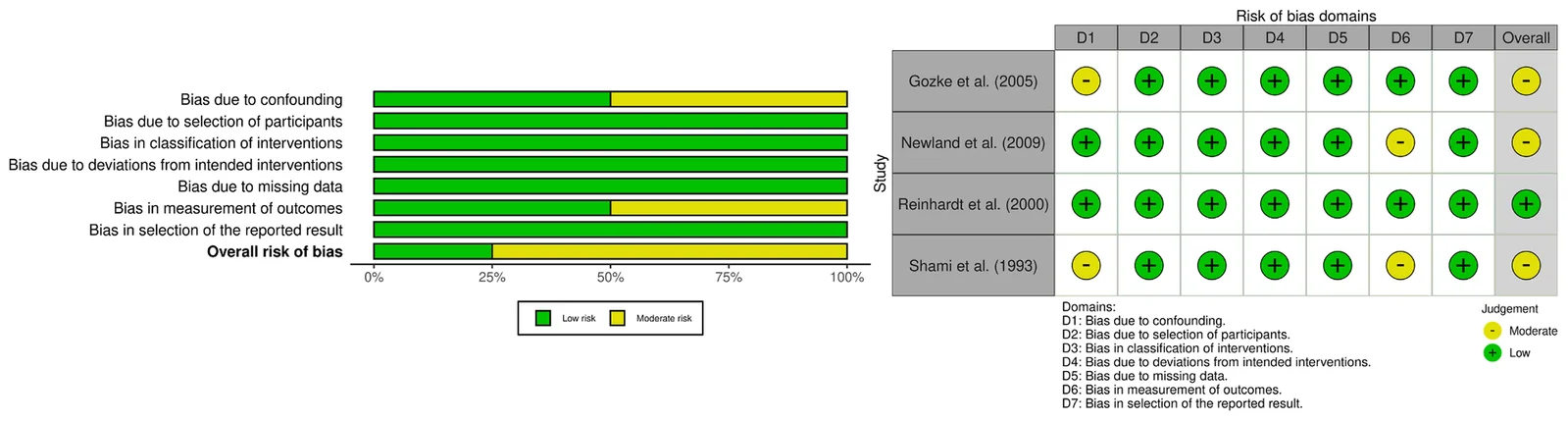
Summary plot and traffic light diagram for the nonrandomized case-control studies assessed using the Risk Of Bias In Non-randomized Studies—of Interventions (ROBINS-I) tool.

**Fig 3:**
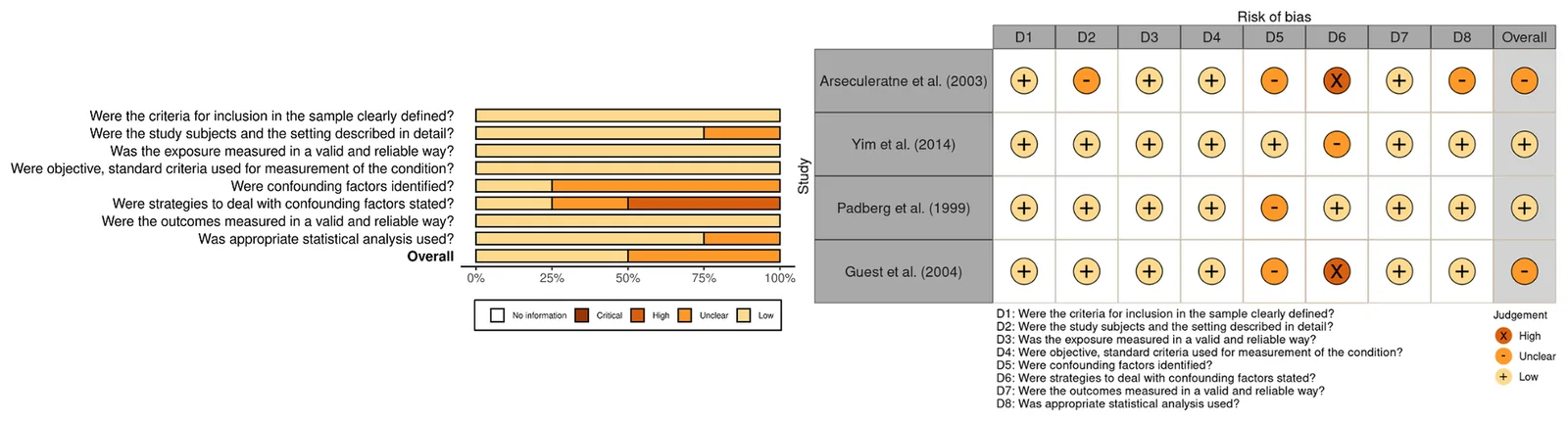
Summary plot and traffic light diagram for the cross-sectional studies.

Fig 2 shows the visual summaries of the ROBINS-I assessments for the case-control studies included in this review. Fig 3 shows the visual summaries of the Joanna Briggs Institute checklist for the cross-sectional studies. Further details of assessments of each domain are provided in the Supplementary Material (online only). Overall, most of the studies had bias due to confounding factors as five studies had residual confounding. Common potential confounders that were not adjusted for included alcohol consumption, body mass index, level of physical activity, nutritional status, and occupational exposures. These are risk factors for peripheral neuropathy and is an important limitation as it may distort the observed association between CVD and neuropathy. Two studies had bias in the measurement of outcomes because it was not stated if the investigators were blinded to CVD status when doing the tests, which could have influenced their interpretation of the participants' responses. One study had bias as the study participant demographics were not described and there was no hypothesis testing. The purpose of this review was to highlight a potential association between CVD and neuropathy, which requires further exploration; we decided because of the limited literature and the qualitative nature of this review that our conclusions will not be weighted by risk of bias, further necessitating cautious interpretation of the findings.

### Methods of neuropathy testing used

The included studies performed a range of tests to assess peripheral nerve function with regards to sensory and motor functions. The methods of neuropathy assessment included various sensory tests, tendon reflex examinations, NCS, and histological studies. Some studies carried out neuropathy testing using the NDS, which is a composite score based on ankle reflex, pinprick sensation, temperature sensation, and vibration perception.^22^ A study assessed sensory thresholds using a pressure aesthesiometer consisting of filaments that buckled under increasing pressure applied to different parts of the limb commonly subjected to skin changes in CVD. Similarly, another study investigated sensory thresholds using a 10-g Owen Mumford monofilament on different areas of the plantar and dorsal aspect of the foot. NCS were used to assess motor and sensory neuropathy by investigating distal motor latency—the time taken from the stimulation of a nerve to the muscle response, compound muscle action potential (CMAP)—the combined electrical potentials of the muscle fibers that are innervated by the motor nerve, and motor conduction velocity of the tibial, sural, and peroneal nerves. Finally, a study used immunohistochemistry of punch biopsies taken immediately adjacent and proximal to the chronic and healing venous ulcer to investigate sensory neuropathy. It looked at substance P (SP) and calcitonin gene-related peptide (CGRP)—both released from C-nerves, and protein gene product 9.5 (PGP 9.5), which is a protein used as a marker for all cutaneous nerves, including cholinergic, adrenergic, and peptidergic nerves.^23^ Further details on neuropathy assessment methods and their findings from each paper are found in Table III.

**Table III:** Neuropathy assessment and findings

Studies	Types of neuropathy assessed	Methods of neuropathy assessment	Nerve fibers affected	Key findings
Arseculeratne et al^16^	Sensory	10-g monofilament	Not stated	53% of patients had some degree of sensory loss in the foot that the ulcer affected. 20% of the sample had more than 50% of foot sensory loss. As the ulcer duration increased, the degree of neuropathy also increased (positive correlation, r^2^ = 0.66).
Gozke et al^8^	Motor and sensory	Electromyography	A-alpha fibers A-beta fibers	Demonstrated a significant decrease in peroneal nerve CMAP and deceleration in tibial nerve motor conduction velocity in patients with CVD. A negative correlation was found between increasing degrees of reflux detected on venous Doppler ultrasound and peroneal nerve CMAP amplitude. As the degree of venous reflux increased, there was a significant prolongation in distal motor latency. There was a highly significant loss of the sural sensory nerve action potential in patients with venous disease.
Guest et al^17^	Peripheral	Punch biopsy and immunostaining for PGP 9.5, CGRP, substance P, and CD34 Image analysis and fiber quantification	C-fibers	Immunohistochemistry showed that there is reduced cutaneous innervation, especially of CGRP positive fibers in patients with venous ulcers. Healing ulcers showed nerve loss in the dermis and nonhealing ulcers showed nerve loss in the epidermis. Significantly reduced CGRP in the dermis of healing and nonhealing ulcers, and was almost completely absent in the epidermis of all ulcers. This is evidence of C-fiber neuropathy which could affect healing of ulcers.
Newland et al^18^	Sensory	NDS: Sensory testing using a 10-g monofilament Vibration perception using a biothesiometer and a 128 Hz tuning fork Temperature discrimination Achilles tendon reflex examination	Not stated	Evidence showed that NDS was significantly worse in patients with venous disease.
Padberg et al^19^	Sensory	Deep tendon reflexes Vibratory, proprioceptive, and light touch sensations tested Motor strength tested	A-alpha fibers A-beta fibers A-delta fibers	Demonstrated significantly increased sensory thresholds at the most common sites of venous ulceration (just proximal to the medial malleolus) between mild and severe cases of CVD. The sensory loss did not follow nerve distributions, but it did match the extent of skin trophic changes. In severe compared with mild cases of venous disease, there was decreased vibration sense, light touch, patellar deep tendon reflex, and Achilles deep tendon reflex. There was no difference in motor strength or proprioception between mild and severe CVD.
Reinhardt et al^7^	Motor and sensory	Motor and sensory nerve conduction studies Vibration testing Thermotesting Quantitative sudomotor axon-reflex test	A-alpha fibers A-beta fibers A-delta fibers C-fibers	In patients with CVD, there is a statistically significant prolongation of distal motor latency of the peroneal nerve. There were significantly increased thresholds for warm and cold detection. A significantly reduced vibration sense was shown in patients with CVD. There were no abnormalities in patients with CVD on evaluation of sudomotor and vasomotor function.
Shami et al^11^	Sensory	Thresholds to warming, cooling, and vibration	C fibers A-delta fibers	There were significantly increased thresholds to warming and vibration in patients with CVD compared with healthy controls. No statistically significant difference between thresholds to cooling for the two groups.
Yim et al^10^	Sensory	NDS: Sensory testing using a 10-g monofilament Vibration perception threshold Temperature discrimination Achilles tendon reflex examination	Not stated	NDS was found to be significantly worse in patients with severe CVD compared with mild CVD. Mild patients with CVD had a mean NDS of 1.00. Severe patients with CVD had a mean NDS of 4.96. Patients with severe CVD had reduced vibration sense, and warmth and cold perception. Analysis showed a negative correlation with increasing ROAM and NDS. Limbs with reduced ROAM for combined plantarflexion and dorsiflexion, as well as limbs with reduced inversion-eversion had higher NDS values.

*CD34*, Cluster determinant 34; *CGRP*, calcitonin gene-related protein; *CMAP*, compound muscle action potential; *CVD*, chronic venous disease; *NDS*, neuropathy disability score; *PGP*, protein gene product; *ROAM*, range of ankle movement.

### Aspects of neurology tested

Six papers tested sensory function, and two papers tested motor function. Sensory function testing was done by vibration testing, temperature perception, monofilament tests, and NCS. From the two studies that used the NDS, one of them performed testing on six sites of the feet in patients with active noninfected venous ulcers or patients who had a history of this (C5 and C6 diseases).^18^ The second study tested neuropathy away from the ulcer site with the sensory testing and vibration perception threshold testing performed at the apex of the first toe, temperature discrimination was tested at the dorsum of the foot, and the Achilles tendon reflex performed at the back of the ankle.^10^ Mean NDS was significantly worse in patients with venous disease compared with healthy controls, and NDS was significantly worse in patients with severe CVD (C5-C6) compared with mild CVD (C1-C2). This study did not assess patients with C3 or C4 stages, and no statistical comparison was done between the individual CEAP classes. Another paper carried out sensory testing on five sites on the limb, including the most common site of venous ulceration – immediately proximal to the medial malleolus.^19^ These sites were assessed as these are common areas, which are subjected to skin changes in CVD. This study excluded patients with C6 classes of CVD as the open ulceration would have made it difficult to assess. Sensory thresholds were significantly worse in patients with severe CVD (C5) compared with mild CVD (C2), with vibration sense, Achilles and patellar deep tendon reflexes, and light touch/pinprick sensations also being diminished. In addition, patients with CVD were found to have significantly increased threshold to warming and cold detection.^7^^,^^11^ NCS were also able to show a significant loss of the sural nerve action potential in patients with CVD.^8^

Papers assessing motor function did this by using NCS. Motor neuropathy was also found to be present in patients with CVD since there was a statistically significant prolongation of the distal motor latency of the peroneal nerve,^7^ a significantly decreased CMAP in the common peroneal nerve, and a deceleration in the tibial nerve motor conduction velocity.^8^

The study that used immunohistochemistry showed that there was a significant decrease in PGP 9.5 and CGRP positive fibers in skin adjacent to venous ulcers, especially in chronic nonhealing ulcers compared with site-matched controls of patients with varicose veins but no skin changes. SP fibers were low in all groups; this was likely due to the age of the participants as neuropeptides levels decrease with age. There was increased dermal microvasculature, shown by CD34 staining, in the ulcer groups, which suggest abnormal dilation of capillaries in patients with CVD. This demonstrated that there was a depletion of neuropeptides in the dermis and epidermis and therefore evidence of C-fiber neuropathy in patients with venous ulcers, which may affect their healing.^17^

### Main findings

Across all the studies, there is increased prevalence of neuropathy in patients with CVD. Mean NDS were significantly worse in patients with venous disease compared with healthy controls.^18^ A study investigated neuropathy in patients with unilateral CVD using a 10-g Owen Mumford monofilament on different areas of the foot, which showed that neuropathy was greater in the CVD affected leg, as 53% of patients had sensory loss in the foot affected by ulcer. Some patients had sensory loss in both legs, but it was more severe in the ulcerated leg.^16^

Some studies showed an association of worsening neuropathy with more severe stages of CVD. There was a trend toward increasing NDS as CEAP class increased with C1 having a mean NDS of 0.71 ± 1.81, C2 being 1.18 ± 2.13, C5 being 3.50 ± 2.38, and C6 being 5.21 ± 2.26.^10^ Similarly, another study stratified patients according to the degree of reflux detected on a venous duplex ultrasound guidance into grade 0, 1, and 2 reflux. As the grade of reflux increased, there was significant prolongation of peroneal nerve distal motor latency, with duplex grade 0 having a mean of 3.7 ± 0.4 ms, grade 1 being 4.6 ± 0.4 ms, and grade 2 being 6.1 ± 1.0 ms, *P* < .0001. In addition, as the reflux increased, the peroneal nerve CMAP amplitude decreased, with grade 0 having a mean of 4.5 ± 4.6 mV, grade 1 being 2.8 ± 13.4 mV, and grade 2 being 2.2 ± 1.8 mV, *P* < .05.^8^ This suggests a defect in the A-alpha nerve fibers because an increase in venous stasis due to more reflux leads to more axonal deterioration. Significant values from each paper can be found in Table IV.

**Table IV:** Significant values from each paper

Study	Statistically significant findings
Arseculeratne et al^16^	Not stated
Gozke et al^8^	Common peroneal nerve mean CMAP amplitude (mV) decreased in patients with CVD (3.6 ± 3.6) compared with healthy controls (5.1± 2.3), *P* < .05. Tibial nerve mean MCV (m/s) decreased in patients with CVD (43.9 ± 6.1) compared with healthy controls (46.7 ± 4.1), *P* < .05. Detectable sural nerve SNAP in patients with CVD (44.6%) was significantly less than in the controls (93.3%). As the grade of reflux detected with venous Doppler ultrasound increased, there was a significant prolongation of the DML of the peroneal nerve, *P* < .0001 and decrease in CMAP amplitude, *P* < .05.
Guest et al^17^	In the dermis adjacent to healing ulcers, the median number and length of fibers is decreased compared with varicose vein controls (25 vs 75, *P* = .033; 827 vs 2242 μm, *P* = .04). Reduction is the number and length of epidermal fibers in the nonhealing ulcer group compared with controls (4 vs 11.5, *P* = .026; 102 vs 225 μm, *P* = .047). Shorter PGP-positive fibers in the dermis of both ulcer groups (healing and nonhealing), with a greater reduction in nonhealers (22 vs 17 μm-nonhealers, 28 μm controls; *P* = .027 and .002, respectively). Shorter CGRP-positive fibers in the dermis of both ulcer groups and in the epidermis of the healing group (1198 μm-controls, 431 μm-healers, 686 μm-nonhealers; *P* = .002 and .04, respectively). Epidermal fiber lengths were shorter in both groups compared with controls (19 μm-controls, 0 μm-healers, 0 μm-nonhealers; *P* < .001) Increased in the total area of CD34-immunostained vessels in both ulcer groups compared with controls (846 μm^2^-controls, 1943 μm^2^-healers, 1715 μm^2^-nonhealers; *P* < .001).
Newland et al^18^	Mean NDS was higher in patients with venous disease (3.3 ± 2.06) compared with healthy controls (0.00 ± 0.00), *P* < .001.
Padberg et al^19^	A significant difference was found between sensory thresholds in limbs with CEAP 2 compared with CEAP 5 at the following areas: proximal to the medial malleolus, proximal to the lateral malleolus, the proximal medial and lateral calf, and thigh. Proximal to the medial malleolus: CEAP 2, 3.61 (2.44-4.17) vs CEAP 5, 4.65 (4.08-6.65); *P* = .00001 Proximal to the lateral malleolus: CEAP 2, 3.84 (2.44-4.17) vs CEAP 5, 4.45 (3.84-6.45); *P* = .0039 Proximal medial calf: CEAP 2, 3.22 (2.36-4.08) vs CEAP 5, 4.24 (3.22-4.94); *P* = .00021 Proximal lateral calf: CEAP 2, 3.22 (2.36-4.17) vs CEAP 5, 4.17 (3.22-5.18); *P* = .007 Thigh: CEAP 2, 2.83 (2.44-4.17) vs CEAP 5, 3.96 (2.36-6.10); *P* = .007. In CEAP 5 vs CEAP 2: Decreased vibration sense (*P* = .0014) Decreased light touch/pinprick (*P* = .0022) Decreased patellar deep tendon reflex (*P* = .0124) Decreased Achilles deep tendon reflex (*P* = .0078)
Reinhardt et al^7^	Prolongation of the DML of the peroneal nerve in patients with CVD (median, 5.4 ms) compared with healthy controls (median, 4.5 ms), *P* = .02. Increased threshold to warming in patients with CVD (median, 9.6 °C) compared with controls (median, 5.2 °C), *P* = .016. Diminished cold perception in patients with CVD (median, 3.45 °C) compared with controls (median, 1.55 °C), *P* = .016. Reduced vibration sense in patients with CVD (median, 2.8925) compared with controls (median, 1.1075), *P* < .008.
Shami et al^11^	Patients with CVD had a raised median threshold to warming, 5.3 °C (0.1-9.1 °C), compared with 1.21 °C (0.17-3.5 °C) in the healthy controls, *P* = .005. Patients with CVD had a reduced median vibration sense, 22 (13-31), compared with the controls, 12 (8.5-27.5), *P* = .024.
Yim et al^10^	Patients with mild CVD (C1-C2) had a lower mean NDS, 1.00 ± 2.00, than patients with severe CVD (C5-C6), 4.96 ± 2.32, *P* < .001.

*CEAP*, Clinical-Etiology-Anatomy-Pathophysiology; *CGRP*, calcitonin gene-related peptide; *CMAP*, Compound muscle action potential; *CVD*, chronic venous disease; *DML*, distal motor latency; *MCV*, motor conduction velocity; *NDS*, Neuropathy Disability Score; *PGP*, protein gene product; *SNAP*, sensory nerve action potential.

### Nerve fibers

Nerve fibers affected in patients with CVD-associated neuropathy were C, A-alpha, A-beta, and A-delta. A-alpha and A-beta nerve fibers were found to be defected through electromyography, vibratory sensation testing, and light touch sensation testing.^7^^,^^8^^,^^19^ C-fiber neuropathy was detected through immunohistochemistry and thermotesting.^7^^,^^11^^,^^17^ A-delta nerve fiber dysfunction was found through investigating the threshold to cooling in patients with CVD.^7^ Unmyelinated C-fibers are used for warm stimuli and nociceptive stimuli perception. Large, myelinated A-beta and A-alpha fibers are involved in the sensation of light touch and vibration sensing. Small, myelinated A-delta nerve fibers mediate non-nociceptive cool stimuli.^24^ The most prevalent symptom of neuropathy was reduced vibratory perception, with four studies providing evidence of this finding. Sensory neuropathy was more frequently reported than motor neuropathy; this could be due to the fact that motor function was not assessed in every included study, as opposed to sensory neuropathy being more prevalent.

## Discussion

This systematic review has reported that there may be evidence of peripheral neuropathy in patients with CVD; however, direct comparisons between studies may not be meaningful because of the heterogeneity of assessment methods and study populations. Although many studies did not report overall prevalence rates of neuropathy in CVD, Reinhardt et al^7^ found that 33.3% of participants studied had either a reduction of the triceps reflex, sensory disturbances, or weakness of distal muscles. Newland et al^18^ showed that 100% of patients with CVD had subjective, objective, and physical signs of neuropathy to a greater degree than healthy controls. In addition, Arseculeratne et al^16^ found that 53% of patients had a loss of sensation in the foot bearing the ulcer, showing a degree of neuropathy. Some studies propose that neuropathy is a factor that increases the risk of ulceration in CVD as it can cause reduced sensory perception, which may lead to damage to skin, breakdown, and ulcer formation. However, this needs to be validated further through longitudinal studies with larger sample sizes. If an association is established, patients with CVD could undergo neuropathy screening to aid early detection of this potential risk factor for ulceration and possibly reduce risk by ensuring that patients receive early superficial venous intervention, compression therapies, and lifestyle changes such as increased exercise.^16^^,^^18^

Guest et al^17^ investigated skin biopsies from patients with VLU compared to those with varicose veins, using immunostaining of abundant neuropeptides found in the skin, SP, CGRP, and PGP 9.5. SP and CGRP are simultaneously released from nociceptive C-fibers, which are known to be affected in ulceration.^11^ Neuropeptides can be neurotransmitters or hormones and are stored in secretory vesicles in nerve terminals, from which they are released during high-frequency nerve stimulation.^25^ A significant decrease in PGP 9.5 and CGRP fibers, and an increased dermal microvasculature were found in patients with chronic ulcers that had failed to heal by 6 months. When peripheral nerves are damaged, there may be dysregulated neuropeptide release – as indicated by the reduced PGP 9.5 and CGRP fibers. This can impair inflammatory signaling, angiogenesis, and keratinocyte proliferation, which may explain the delayed wound healing in chronic ulcers.^26^ This allows the postulate that the decrease in neuropeptides and the increase in epidermal nerve fibers in chronic ulcers may be involved in healing.

Peripheral nerves need to have a sufficient oxygen supply to function correctly. The endoneurium, a connective tissue surrounding axons in peripheral nerves, has a supply of microvessels providing oxygen alongside the arteries and capillaries that supply the nerves. CVD can lead to venous hypertension and increased inflammatory factors. Venous microangiopathy occurs when venous hypertension and stasis cause microvascular changes. Blood and lymphatic capillaries become dilated, elongated, and tortuous, with large fenestrae that cause an increased amount of pericapillary leakage, impairing blood flow to nerves and leading to endoneurial hypoxia.^7^^,^^8^^,^^11^^,^^15^ This can also lead to edema in the fascicles of peripheral nerves and increased endoneurial pressure, which can compress and destroy axons causing axonal ischemia, resulting in neuropathy.^7^^,^^11^^,^^16^^,^^17^ One study suggests that the decreased nerve conduction, vibration sense, pinprick sensitivity, and temperature perception in patients with CVD is attributable to nerve ischemia due to venous microangiopathy.^10^

There is an inflammatory response to venous hypertension as there are pericapillary fibrin cuffs, formed by capillaries leaking fibrinogen into surrounding tissue, and leukocyte trapping and activation. Studies hypothesize that this may affect the peripheral nerve trunks in limbs of patients with worse stages of CVD.^10^^,^^19^ These cuffs cause dysfunction of peripheral nerves as they obstruct the exchange of oxygen, nutrients, and growth factors. These are thought to lead to the breakdown of tissue and ulceration.^10^ Two of the included studies required the participants to undergo compression therapy to reduce inflammation before neurophysiological examinations, with one study explicitly stating stockings were applied at least 6 hours before. This was done to prevent edema influencing the reliability of the conduction studies.^7^^,^^8^

Venous stasis, a hypercoagulable state, and vein damage all contribute to the development of venous thrombosis in patients with CVD.^15^ The microvascular thrombosis theory suggests that the repeated formation of microthrombi cause capillary occlusion, which reduce oxygen and nutrient access to the skin. This blockage may cause tissue hypoxia and inflammation, which can reduce the number of capillaries seen in severe cases of CVD. This could be a factor in neuropathy development as it results in tissue ischemia and necrosis.^7^^,^^16^^,^^27^

An alternative hypothesis is that a depletion of neuropeptides from the nerve endings could be the cause of neuropathy in CVD.^11^^,^^17^ They aid the functioning of the dermal microvasculature by regulating blood flow through vasodilation and vasoconstriction.^28^ Although walking, there is a requirement to vasoconstrict in response to an increase in venous pressure through sympathetic activation, known as the venoarteriolar reflex. In patients with venous hypertension, a constant requirement for sustained sympathetic activation may lead to a depletion of neuropeptides from the nerve endings. With little time for recovery or manufacturing of sufficient amounts of neuropeptides, there is an impairment of the venoarteriolar reflex, which can increase edema and limit mobility.^11^

The results from this review can be used to inform clinicians' understanding of venous disease as many patients present with neurological symptoms, but the link between neuropathy and venous disease has not been adequately highlighted, so their symptoms can be attributed to incorrect causes. It has been indicated that there may be a neurotrophic disturbance in CVD that could contribute to the etiology of ulceration, which requires further exploration. The current literature indicates that there is predominantly sensory neuropathy present in patients with CVD, but confirmation of this requires more motor testing using NCS as this was limited. To date, this is the first systematic review to the authors' knowledge that evaluates and synthesizes the current literature on neuropathy prevalence in patients with CVD, providing a reference point for both clinicians and researchers.

As there are only a few studies that have been performed to assess the extent and prevalence of peripheral neuropathy in patients with CVD, some studies included in this review had small sample sizes (10-42 participants) and a lack of representation of different CEAP stages, namely C3 and C4, limiting their generalizability and statistical precision. There was heterogeneity in the study designs and neuropathy assessment methods, which meant comparisons were difficult to make. Some of the papers failed to report the presence of comorbidities that contribute to neuropathy in the participants, as well as the use of compression therapy, which affects peripheral nerve function. This inconsistent reporting of variables is an important point that may have introduced bias into the studies included in this systematic review. Conditions such as heart failure and liver failure can cause leg edema, which is associated with neuropathy; however, they may not traditionally be thought of as diseases that cause neuropathy so were not excluded. Factors such as age, body mass index, alcohol consumption, physical activity level, and nutritional status were not adjusted for which led to some studies having residual confounding. This affects the conclusions, as these are risk factors for peripheral neuropathy and could therefore distort the observed association between CVD and neuropathy. There also may have been outcome measurement bias due to lack of blinding of investigators to CVD status in two of the studies. This is essential to be addressed in future research to accurately determine what degree of neuropathy is due to CVD and not confounding factors. The quality assessment of these studies is an important factor, as most of the studies had a low-moderate risk of bias, so the findings of this review must be interpreted cautiously with an explanatory focus rather than confirmatory. A sensitivity analysis was not possible because of small number of studies with heterogeneous methods. Five studies investigated sensory thresholds in the feet of patients with CVD, and two studies tested these in the gaiter areas. The two studies that carried out NCS examined the head of the fibula, ankle, and popliteal fossa for the peroneal nerve, the lateral malleoli, and the area over the triceps surae muscle for the sural nerve. Considering that neuropathy assessment mainly looked at the foot, the anatomical distribution of neuropathy in CVD cannot be accurately determined. Future work can look to compare the severity of neuropathy in the feet and gaiter regions. Furthermore, it was not stated in some of the papers whether they avoided scar tissue, this should be explicitly established to know whether testing was influenced by skin changes rather than neuropathy alone. Finally, there was lack of geographical representation as most studies included originated from England or the United States.

This review highlights the need for larger, adequately powered, longitudinal, prospective studies that can further investigate the occurrence of neuropathy in CVD and explore at exactly what stage of CVD does it start to develop. Disparate methods used also reveals the importance of standardizing the method of neuropathy assessment tools for these studies in the future to better analyze results across papers. Future studies could investigate whether objective neuropathy findings correlate with histopathological findings and further detect whether there is reversibility of this after treatment of the venous insufficiency. In addition, more mechanistic studies could be carried out investigating the effect of venous microangiopathy on nerve function to identify the exact cause of neuropathy in patients with CVD.

## Conclusions

The included studies showed evidence of either sensory or motor neuropathy in patients with CVD, with features of neuropathy demonstrated in between 33% and 100% of participants; however, direct comparability is greatly limited because of heterogeneous assessment methods and lack of pooled confidence intervals. There are several proposed mechanisms that may lead to this finding. Endoneural hypoxia and chronic ischemia from venous microangiopathy may occur due to venous hypertension and subsequent microvascular changes. This potentially results in demyelination and axonal degeneration, leading to neuropathy. In addition, depletion of neuropeptides due to constant sympathetic activation is another potential mechanism of neuropathy in patients with CVD. This review has highlighted a link, which should be investigated further to evaluate the role of neuropathy in venous disease. Future work is required to determine the temporal relationship between CVD progression and neuropathy development to see if this is related to the risk of disease progression, standardize neuropathy assessment in venous disease, identify the subgroups of patients that neuropathy assessment would be clinically useful in, and increase investigation of motor function to allow conclusive comparisons to be made to sensory neuropathy.

## Author Contributions

Conception and design: VR, JB, MS, MT, RL, SO, AD

Analysis and interpretation: VR, JB, MS, MT

Data collection: VR, JB, MS, MT

Writing the article: VR

Critical revision of the article: VR, JB, MS, MT, RL, SO, AD

Final approval of the article: VR, JB, MS, MT, RL, SO, AD

Statistical analysis: Not applicable

Obtained funding: Not applicable

Overall responsibility: AD

## Funding

None.

## Disclosures

None.
