# Supplementary material for: A systematic review exploring the link between chronic venous disease and neuropathy
Source: J Vasc Surg Venous Lymphat Disord. 2026 Aug 19;14(6):102602. doi: 10.1016/j.jvsv.2026.102602 (PMC13598183; doi:10.1016/j.jvsv.2026.102602)
Supplement: Supplementary file 1 [file mmc1.docx]

**Supplementary Material**

**Full Searches:**

MEDLINE: searched 15/08/2025 12:22 pm

Ovid MEDLINE(R) ALL <1946 to August 14, 2025>

1	exp Varicose Veins/	20174

2	Venous Insufficiency/	7361

3	Venous insufficienc*.ti,ab.	5835

4	Vein insufficiency.ti,ab.	283

5	Venous disease*.ti,ab.	3470

6	Venous disorder*.ti,ab.	661

7	Venous valvular.ti,ab.	165

8	Vein disorder*.ti,ab.	24

9	Vascular Diseases/	40250

10	Neuropath*.ti,ab.	174042

11	Nerve disease*.ti,ab.	1312

12	Nerve damage*.ti,ab.	8582

13	Nerve disorder*.ti,ab.	1226

14	Nerve dysfunction*.ti,ab.	2996

15	Neuromuscular disease*.ti,ab.	7953

16	Peripheral nervous system disease*.ti,ab.	200

17	1 or 2 or 3 or 4 or 5 or 6 or 7 or 8 or 9	67757

18	10 or 11 or 12 or 13 or 14 or 15 or 16	192363

19	17 and 18	460

20	limit 19 to (english language and humans)	360

EMBASE: searched 15/08/2025 12:36 pm

Embase Classic+Embase <1947 to 2025 August 13>

1	varicosis/	36665

2	vein insufficiency/	8461

3	exp chronic vein insufficiency/	5350

4	vein insufficienc*.ti,ab.	530

5	venous insufficienc*.ti,ab.	9615

6	venous disease*.ti,ab.	5635

7	Venous disorder*.ti,ab.	1055

8	Venous valvular.ti,ab.	242

9	vein disorder*.ti,ab.	51

10	vascular disease/	86905

11	venous reflux/	3108

12	venous stasis/	3578

13	neuropathic pain/	50238

14	peripheral neuropathy/	64250

15	nerve damage*.ti,ab.	12587

16	nerve disease*.ti,ab.	1898

17	nerve disorder*.ti,ab.	1791

18	nerve dysfunction*.ti,ab.	4055

19	neuropathy/	63946

20	neuromuscular disease/	24577

21	neuropath*.ti,ab.	265040

22	1 or 2 or 3 or 4 or 5 or 6 or 7 or 8 or 9 or 10 or 11 or 12	142299

23	13 or 14 or 15 or 16 or 17 or 18 or 19 or 20 or 21	368142

24	22 and 23	3467

25	limit 24 to (human and english language)	2673

26	limit 25 to conference abstract status	559

27	25 not 26	2114

Cochrane CENTRAL: searched 15/08/2025 12:47 pm **36 trials**

Search Name:

Cochrane CENTRAL search

Last Saved:	15/08/2025 12:46:55

Comment:

ID	Search

#1	(venous NEXT insufficienc*):ti,ab,kw (Word variations have been searched)

#2	(vein NEXT insufficienc*):ti,ab

#3	(venous NEXT disease*):ti,ab

#4	(venous NEXT disorder*):ti,ab

#5	(vein NEXT disorder*):ti,ab

#6	(vascular NEXT disease*):ti,ab

#7	(neuropath*):ti,ab

#8	(nerve NEXT damage*):ti,ab

#9	(nerve NEXT disorder*):ti,ab

#10	(nerve NEXT dysfunction*):ti,ab

#11	(neuromuscular NEXT disease*):ti,ab

#12	(vein*):ti,ab

#13	(venous):ti,ab

#14	MeSH descriptor: [Venous Insufficiency] explode all trees

#15	MeSH descriptor: [Varicose Veins] explode all trees

#16	MeSH descriptor: [Peripheral Nervous System Diseases] explode all trees

#17	#1 OR #2 OR #3 OR #4 OR #5 OR #14 OR #15 OR ((#12 OR #13) AND #6)

#18	#7 OR #8 OR #9 OR #10 OR #11 OR #16

#19	#17 AND #18

**Risk of Bias Assessment:**

**Supplementary Table 1:** Risk of bias analysis of all non-randomised case-control studies (ROBINS-I tool).

| Study | **D1** | **D2** | **D3** | **D4** | **D5** | **D6** | **D7** | **Overall** |
| --- | --- | --- | --- | --- | --- | --- | --- | --- |
| Gozke et al. (2005) | Moderate | Low | Low | Low | Low | Low | Low | Moderate |
| Newland et al. (2009) | Low | Low | Low | Low | Low | Moderate | Low | Moderate |
| Reinhardt et al. (2000) | Low | Low | Low | Low | Low | Low | Low | Low |
| Shami et al. (1993) | Moderate | Low | Low | Low | Low | Moderate | Low | Moderate |

**Supplementary Table 2:** Risk of bias analysis of cross-sectional studies (Joanna Briggs Institute Critical Appraisal Checklist for Analytical Cross-Sectional Studies).

| **Criteria** | Arseculeratne et al. (2003) | Yim et al. (2014) | Padberg et al. (1999) | Guest et al. (2004) |
| --- | --- | --- | --- | --- |
| 1. **Were the criteria for inclusion in the sample clearly defined?** | Yes | Yes | Yes | Yes |
| 2. **Were the study subjects and the setting described in detail?** | Partially | Yes | Yes | Yes |
| 3. **Was the exposure measured in a valid and reliable way?** | Yes | Yes | Yes | Yes |
| 4. **Were objective, standard criteria used for measurement of the condition?** | Yes | Yes | Yes | Yes |
| 5. **Were confounding factors identified?** | Partially | Yes | Partially | Partially |
| 6. **Were strategies to deal with confounding factors stated?** | No | Partially | Yes | No |
| 7. **Were the outcomes measured in a valid and reliable way?** | Yes | Yes | Yes | Yes |
| 8. **Was appropriate statistical analysis used?** | Partially | Yes | Yes | Yes |
| **Overall Risk of Bias** | Moderate | Low | Low | Moderate |
